# High-Performance GaN-Based Green Flip-Chip Mini-LED with Lattice-Compatible AlN Passivation Layer

**DOI:** 10.3390/nano15131048

**Published:** 2025-07-05

**Authors:** Jiahao Song, Lang Shi, Siyuan Cui, Lingyue Meng, Qianxi Zhou, Jingjing Jiang, Conglong Jin, Jiahui Hu, Kuosheng Wen, Shengjun Zhou

**Affiliations:** 1 Center for Photonics and Semiconductors, School of Power and Mechanical Engineering, Wuhan University, Wuhan 430072, China; jiahaosong@whu.edu.cn (J.S.); shilang@whu.edu.cn (L.S.); cuisiyuan@whu.edu.cn (S.C.); 2024202080001@whu.edu.cn (L.M.); qianxizhou@whu.edu.cn (Q.Z.); 2 Wuhan JingWei Technology Co., Ltd., Wuhan 430070, China; jj_jiang@wh-jingwei.com; 3 Jiangxi SMTC Semiconductor Co., Ltd., Nanchang 330096, China; jinconglong@szmtc.com.cn (C.J.); hujiahui@szmtc.com.cn (J.H.); wenkuosheng@szmtc.com.cn (K.W.)

**Keywords:** green flip-chip mini-LED, atomic layer deposition, passivation layer, AlN, external quantum efficiency

## Abstract

The GaN-based green miniaturized light-emitting diode (mini-LED) is a key component for the realization of full-color display. Optimized passivation layers can alleviate the trapping of carriers by sidewall defects and are regarded as an effective way to improve the external quantum efficiency (EQE) efficiency of mini-LEDs. Since AlN has a closer lattice match to GaN compared to other heterogeneous passivation materials, we boosted the EQE of GaN-based green flip-chip mini-LEDs through the deposition of a lattice-compatible AlN passivation layer through atomic layer deposition (ALD) and a SiO_2_ passivation layer through plasma-enhanced chemical vapor deposition (PECVD). Benefiting from reduced sidewall nonradiative recombination, the EQE of the green flip-chip mini-LED with a composite ALD-AlN/PECVD-SiO_2_ passivation layer reached 34.14% at 5 mA, which is 34.6% higher than that of the green flip-chip mini-LED with a single PECVD-SiO_2_ passivation layer. The results provide guidance for the realization of high-performance mini-LEDs by selecting lattice-compatible passivation layers.

## 1. Introduction

GaN-based LEDs have attracted considerable attention due to their advantages, including high luminous efficiency, low energy consumption, and long operation lifetime [1,2,3,4,5]. However, due to mismatches in lattice constants and thermal expansion coefficients between GaN and sapphire substrate, there is a large number of threading dislocations in the epitaxial layer of GaN-based LEDs, leading to a decrease in emission efficiency [6]. Another major challenge contributing to efficiency degradation is related to Shockley–Read–Hall (SRH) recombination, which is caused by plasma-induced etching damage. According to previous studies, crystallographic defects, impurities, and dangling bonds were observed on the etched surface, which build trap states within the bandgap and thus act as SRH nonradiative recombination centers, leading to efficiency droop [7,8]. A range of techniques have been used to reduce etching damage, such as thermal annealing, chemical etching, H_2_ plasma treatment, and sidewall passivation layers [9,10,11,12,13,14]. Among the various techniques, sidewall passivation using dielectric materials is arguably the most promising for achieving high-performance mini-LEDs.

A sidewall passivation layer is generally grown by PECVD, which uses hydrogen-containing precursors and provides rapid deposition rates [15]. However, a passivation layer grown by PECVD is unable to completely cover the surface of the mini/micro-LED due to its poor step coverage ability. ALD uses self-limiting interactions between the precursor gas and the substrate surface, and thin films are deposited layer-by-layer through alternately introducing precursor gas [16,17]. Therefore, the growth thickness can be precisely controlled by ALD, which is more effective at depositing dense dielectric films compared to PECVD [18,19]. Heterogeneous materials including SiO_2_, Al_2_O_3_, and HfO_2_ have been adopted in previously reported passivation schemes. Deng et al. investigated the optical and electrical characteristics of green micro-LEDs with ALD-SiO_2_ passivation layers of different thicknesses [20]. Wong et al. used ALD to deposit 50 nm thick SiO_2_ as the passivation layer, achieving micro-LEDs with uniform light emission and low leakage current [21]. Lee et al. investigated the effect of ALD-Al_2_O_3_ (50 nm)/PECVD-SiO_2_ (250 nm) and PECVD-SiO_2_ (300 nm) passivation layers on the leakage current and EQE of GaN-based micro-LEDs [22]. Prechatavanich et al. analyzed the impact of SiO_2_, Al_2_O_3_, and HfO_2_ passivation layers on AlGaInP-based red micro-LEDs [23]. However, from the perspective of crystallography, it is more appropriate to use homogeneous materials or lattice-compatible materials as the passivation layer. A homogeneous GaN passivation layer is theoretically the superior candidate for GaN-based LEDs [24]. However, the low electrical resistivity of intrinsic GaN make it unsuitable as a passivation material in practice. Compared to other heterogeneous passivation layer materials (SiO_2_, Al_2_O_3_, HfO_2_), AlN has a closer lattice match to GaN [25], rendering it a potential passivation layer for GaN-based mini/micro-LEDs.

In this work, we fabricated GaN-based green flip-chip mini-LEDs with different types of passivation layers (PECVD-SiO_2_, ALD-SiO_2_/PECVD-SiO_2_, ALD-AlN/PECVD-SiO_2_) and investigated the impact of deposition methods and passivation materials on the optical and electrical properties of green flip-chip mini-LEDs. By comparing the far-field angular emission pattern and near-field light-emitting intensity distributions of green flip-chip mini-LEDs with different passivation layers, we demonstrate the influence of the film quality and refractive index of the passivation materials on the light extraction efficiency (LEE) of green flip-chip mini-LEDs. Benefiting from reduced sidewall nonradiative recombination, the EQE of the green flip-chip mini-LED with a composite ALD-AlN/PECVD-SiO_2_ passivation layer was increased by 34.6% compared to that of the green flip-chip mini-LED with a single PECVD-SiO_2_ passivation layer.

## 2. Experimental

**Figure 1:**
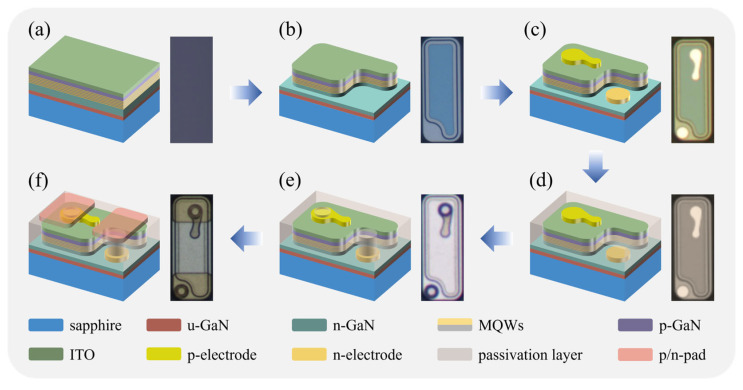
Fabrication steps of the GaN-based green flip-chip mini-LEDs with top-view micro-graphs of the mini-LEDs for each step: (**a**) epilayers of the green flip-chip mini-LEDs; (**b**) defining the mesa structure; (**c**) evaporation of the n-electrode and p-electrode; (**d**) deposition of the SiO_2_ passivation layer and evaporation of DBR; (**e**) formation of the n-electrode and p-electrode holes; (**f**) evaporation of the n-contact and p-contact pads.

GaN-based LEDs were grown on a c-plane patterned sapphire substrate (PSS) by using a metal–organic chemical vapor deposition (MOCVD) system. Trimethylaluminum (TMAl), trimethylindium (TMIn), trimethylgallium (TMGa)/triethylgallium (TEGa), and ammonia (NH_3_) were used as Al, In, Ga, and N sources, respectively. Silane (SiH_4_) and bis (cyclopentadienyl) magnesium (CP_2_Mg) were used for n- and p-type doping, respectively. The LED epitaxial structure consisted of a 20 nm thick sputtered AlN nucleation layer, a 3.5 µm thick undoped GaN buffer layer (temperature = 1125 °C, pressure = 150 Torr, NH_3_ = 65,000 sccm, TMGa = 950 sccm), a 2 µm thick Si-doped n-GaN (temperature = 1100 °C, pressure = 150 Torr, TMGa = 650 sccm, NH_3_ = 45,000 sccm, Si-doping = 1.76 × 10^19^ cm^−3^), 9-period In_0.24_Ga_0.76_N (2.3 nm)/GaN (12 nm) multiple quantum wells (InGaN quantum well: temperature = 720 °C, pressure = 200 Torr, TEGa = 391 sccm, TMIn = 300 sccm, NH_3_ = 67,000 sccm; GaN barrier: temperature = 870 °C, pressure = 200 Torr, TEGa = 332 sccm, NH_3_ = 67,000 sccm), a 20 nm thick p-Al_0.2_Ga_0.8_N electron blocking layer (temperature = 955 °C, pressure = 100 Torr, TMGa = 605 sccm, TMAl = 444 sccm, NH_3_ = 60,000 sccm, Mg-doping = 6.57 × 10^20^ cm^−3^), and a 110 nm thick Mg-doped p-GaN layer (temperature = 965 °C, pressure = 200 Torr, TMGa = 605 sccm, NH_3_ = 60,000 sccm, Mg-doping = 2.89 × 10^20^ cm^−3^). For the fabrication of mini-LEDs, a 110 nm thick indium–tin oxide (ITO) layer was deposited on top of p-GaN via electron beam evaporation, followed by thermal annealing in O_2_ ambience at 550 °C for 20 min [Figure 1a]. The inductively coupled plasma (ICP) etching based on BCl_3_/Cl_2_ mixture gas was used to expose the n-GaN layer and define the mesa [Figure 1b]. Then, Cr/Al/Ti/Pt/Au metallization stacks were deposited on the ITO and exposed n-GaN layer [Figure 1c]. Next, an 800 nm thick SiO_2_ layer was deposited using PECVD, and Ti_3_O_5_ (54.8 nm)/SiO_2_ (89.1 nm) distributed Bragg reflector (DBR) stacks were sputtered on the SiO_2_ layer by electron beam evaporation [Figure 1d]. Afterwards, n- and p-electrode holes were formed by using ICP etching based on CHF_3_/Ar/O_2_ mixture gas [Figure 1e]. Cr/Al/Ti/Pt/Ti/Pt/Au metal stacks were then deposited as the n- and p-contact pads [Figure 1e]. Finally, LED wafers were diced into chips with dimensions of 165 µm × 70 µm. The current–voltage (*I–V*) curves and light output power (LOP) of the mini-LEDs were measured by a Everfine ATA-500 automatic temperature-controlled photoelectric analyzing system. The cross-sectional transmission electron microscopy (TEM) images were taken using FEI Talos F200X at 5 kV.

For brevity, sample A is used to denote the green flip-chip mini-LED with a 920 nm thick PECVD-SiO_2_ passivation layer. Sample B is used to denote the green flip-chip mini-LED with a 120 nm thick ALD-SiO_2_ passivation layer and an 800 nm-thick PECVD-SiO_2_ passivation layer. Sample C is used to denote the green flip-chip mini-LED with a 120 nm thick ALD-AlN passivation layer and an 800 nm thick PECVD-SiO_2_ passivation layer.

## 3. Results and Discussion

**Figure 2:**
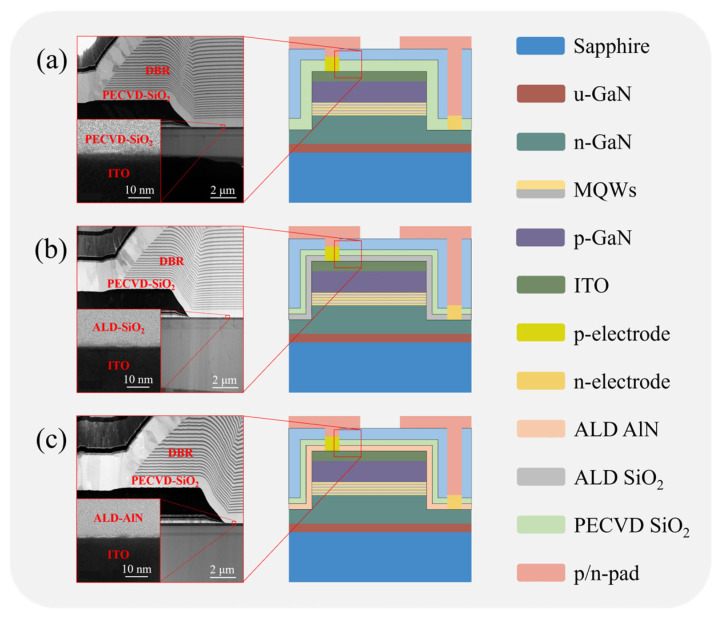
Schematics and cross-sectional TEM images of green flip-chip mini-LEDs with different passivation layers. (**a**) Sample A with single PECVD-SiO_2_ passivation layer; (**b**) sample B with a composite ALD-SiO_2_/PECVD-SiO_2_ passivation layer; (**c**) sample C with a composite ALD-AlN/PECVD-SiO_2_ passivation layer.

We compared the effects of three types of passivation layers on the optical and electrical performances of the green flip-chip mini-LEDs. Figure 2a–c show schematic illustration and cross-sectional TEM images of sample A, sample B, and sample C, respectively. Since it is difficult for electrons to penetrate the metal atoms in ITO, the ITO layer appears as a dark color in the TEM image. In Figure 2a, we can clearly observe a 920 nm thick PECVD-SiO_2_ passivation layer deposited on the top of the ITO layer. The ALD-SiO_2_ and ALD-AlN passivation layers deposited on the top of ITO can be clearly seen in Figure 2b,c. Adjacent to the ALD-SiO_2_ and ALD-AlN passivation layers, an 800 nm thick PECVD-SiO_2_ passivation layer can be obviously seen, as shown in Figure 2b,c. The Ti_3_O_5_/SiO_2_ DBR deposited on top of the PECVD-SiO_2_ passivation layer has a high reflectivity of up to 98.52% in the green wavelength, which can enhance the LEE of the green flip-chip mini-LEDs.

**Figure 3:**
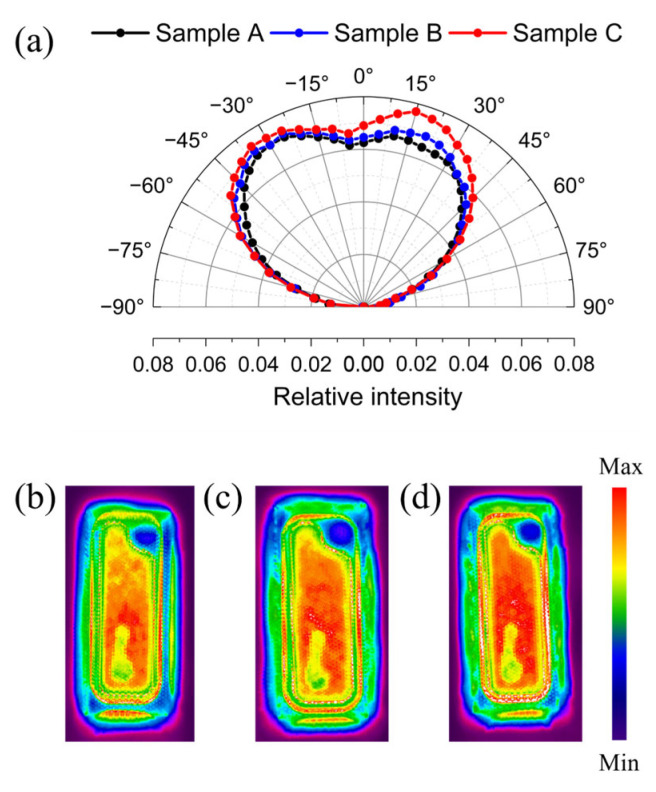
(**a**) Far-field emission pattern of sample A, sample B, and sample C at 5 mA. Near-field light-emitting intensity distributions of (**b**) sample A, (**c**) sample B, and (**d**) sample C at 5 mA.

Figure 3a shows the far-field angular emission pattern of sample A, sample B, and sample C at an injection current of 5 mA. We find that sample B possess a higher emission intensity in comparison with sample A. This result reveals that the passivation effect of ALD-SiO_2_ on the sidewall is more effective in weakening the probability of carriers being captured by defect levels compared to PECVD-SiO_2_, thereby significantly reducing SRH nonradiative recombination and improving emission intensity [26]. Furthermore, it is found that the emission intensity of sample C is significantly improved in the side direction as compared to sample A and sample B. This result indicates that the lattice-compatible AlN passivation layer can more effectively enhance the LEE of the mini-LED compared to the SiO_2_ passivation layer. To further investigate the effects of the three types of passivation layers on the uniformity of the current distribution across InGaN/GaN multiple quantum wells’ (MQWs) active regions, we measured the near-field light-emitting intensity distributions of sample A, sample B, and sample C at 5 mA, as shown in Figure 3b–d. We can observe that sample C shows better uniformity and higher light-emitting intensity compared to sample A and sample B. The related mechanisms can be described as follows.

By neglecting the effects of multiple reflections between the dielectric layers, we can calculate the transmittance of the single SiO_2_ passivation layer and the composite AlN/SiO_2_ passivation layer using the following formula when light exits vertically from the inside of the chip [27]:

(1) $$T_{j}=\prod_{i=1}^{j}\frac{4n_{i}n_{i+1}}{{\left(n_{i}+n_{i+1}\right)}^{2}}$$

where *T* represents the transmittance, *j* = 2 corresponds to the single passivation layer case (SiO_2_), and *j* = 3 corresponds to the composite passivation layer case (AlN/SiO_2_). The refractive indices *n_i_* of GaN, AlN, SiO_2_, and air are 2.46, 2.16, 1.45, and 1.00, respectively. From Equation (1), the Fresnel power transmission can be calculated to be 90.2% for the single SiO_2_ passivation layer and 92.5% for the composite AlN/SiO_2_ passivation layer. Benefiting from higher transmittance, sample C with the composite ALD-AlN/PECVD-SiO_2_ passivation layer has a higher LEE compared to sample A and sample B, which is consistent with the conclusion obtained from Figure 3.

**Figure 4:**
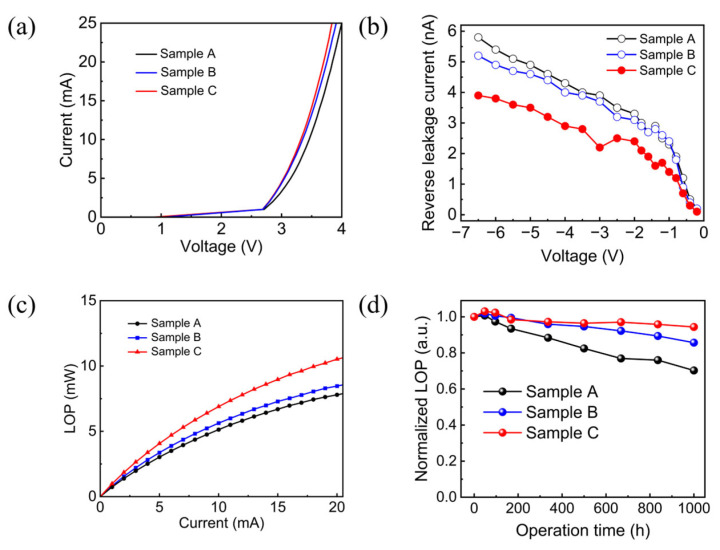
(**a**) I-V characteristics; (**b**) reverse *I–V* characteristics; (**c**) *L–I* characteristics, and (**d**) normalized LOP degradation after prolonged high-temperature and high-humidity aging test of sample A, sample B, and sample C.

Figure 4a,b show the measured forward and reverse *I–V* characteristics of sample A, sample B, and sample C. At 5 mA, the forward voltages of sample A, sample B, and sample C are 3.15 V, 3.04 V and 3.03 V, respectively. Under the same injection current level, the forward voltage of the sample C is slightly lower than those of sample B and sample A. At a negative bias of −5 V, the reverse leakage currents of sample A, sample B, and sample C are 4.9 nA, 4.6 nA and 3.5 nA, respectively, as seen in Figure 4b. The leakage current of sample B is lower than that of sample A, revealing that the ALD-SiO_2_ passivation layer can effectively suppress the leakage current generated by surface defects compared to the PECVD-SiO_2_ passivation layer. This is attributable to the fact that ALD employs metal–organic precursors that are capable of ensuring optimal dielectric quality and yields precise thickness control at the atomic level. Figure 4c shows the LOP versus current characteristics of sample A, sample B, and sample C. Under a 5 mA injection current, the LOP values of sample A, sample B, and sample C are 3.03 mW, 3.37 mW, and 4.07 mW, respectively. The relationship between EQE and the LOP value can be calculated using the following formula:

(2) $$EQE=\frac{Pe}{Ihv}$$

where *P* represents the LOP value, *e* is the elementary charge, *I* is the injection current, *h* is the Planck constant, *v* is the photon frequency. From Equation (2), the *EQE* values of sample A, sample B, and sample C are 25.4%, 28.26%, and 34.14% respectively. Compared to sample A, the EQE of sample B and sample C increased by 7.7% and 34.6%, respectively. This is consistent with the results of the comparison of the near-field light-emitting intensity distributions in Figure 3b,d. Moreover, we investigated the optical degradation of the flip-chip mini-LEDs at a temperature of 85 °C and humidity of 85%, using an injection current of 5 mA. The intervals were fixed at 48h, 96h, 168h, 336h, 500h, 668h, 836h, and 1000h. As shown in Figure 4d, the LOPs of the flip-chip mini-LEDs increase during the initial aging stage, owing to the positive factors of Mg dopant activation and the annealing effect [2]. After 1000 h of aging, the LOPs of sample A and sample B showed degradations of 29.75% and 14.33%, respectively. Conversely, sample C exhibited an exceptional stability in optical performance. This improvement in stability allowed sample C with an ALD-AlN passivation layer to maintain 94.39% of its initial LOP after 1000 h of aging testing. The results indicate that the green flip-chip mini-LED with a lattice-compatible ALD-AlN passivation layer exhibits markedly smaller optical degradation and thus higher device reliability under harsh conditions.

**Figure 5:**
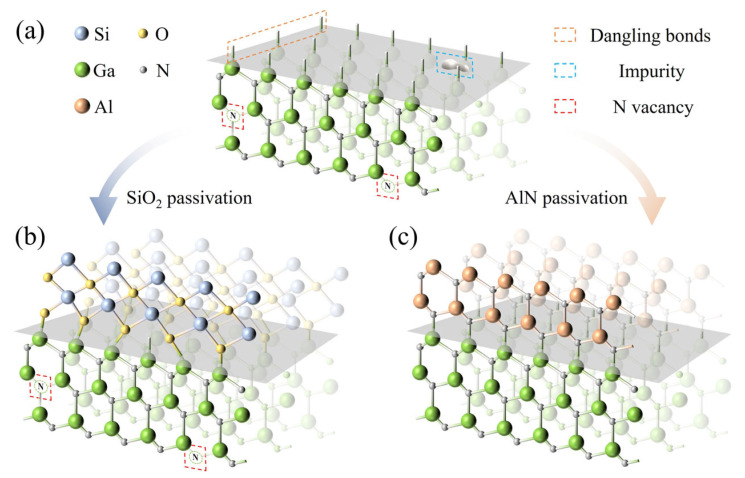
Atomic structure models of the sidewall surface (**a**) before passivation, (**b**) after SiO_2_ passivation, and (**c**) after AlN passivation.

To interpret the difference between the SiO_2_ and AlN passivation layers in reducing the surface state density, atomic structure models of the sidewall surface before and after passivation are shown in Figure 5. After ICP etching, the exposed GaN sidewalls generated a large number of dangling bonds, impurity contaminations, and lattice defects [28,29,30], as shown in Figure 5a. These plasma damages increased the density of surface states, leading to increased SRH recombination rates and severe leakage current [31]. In addition, hybrid density-functional theory (DFT) calculations have demonstrated that N vacancies are the main lattice defects on the sidewalls of GaN-based LEDs and act as a midgap position for the SRH recombination [32]. Using SiO_2_ for sidewall passivation can remove impurity contaminations and saturate partially dangling bonds. However, the lattice mismatch between SiO_2_ and GaN introduces internal stress, and the SiO_2_ passivation layer cannot effectively eliminate surface N vacancies, as shown in Figure 5a. Instead, using AlN for sidewall passivation may be an effective way of eliminating nitrogen vacancies by acting as a nitrogen source [33]. Meanwhile, a lattice-compatible AlN passivation layer can significantly reduce the number of dangling bonds, thereby reducing leakage current and enhancing the stability of green flip-chip mini-LEDs, as shown in Figure 5c.

## 4. Conclusions

In summary, we have demonstrated that the green flip-chip mini-LED with a composite ALD-AlN/PECVD-SiO_2_ passivation layer had a higher light-emitting intensity and lower leakage current compared to the green flip-chip mini-LED with a single PECVD-SiO_2_ passivation layer. Benefiting from reduced sidewall nonradiative recombination, the EQE of the green flip-chip mini-LED with a composite ALD-AlN/PECVD-SiO_2_ passivation layer reached 34.14% at 5 mA, which is 34.6% higher than that of the green flip-chip mini-LED with a single PECVD-SiO_2_ passivation layer. In addition, burn-in tests show the significantly lower optical degradation and thus higher device reliability of the green flip-chip mini-LED with a composite ALD-AlN/PECVD-SiO_2_ passivation layer.

## Data Availability

The data presented in this study are available on request from the corresponding author.
